# A Rare Case of Bilateral Popliteal Artery Occlusion

**DOI:** 10.7759/cureus.20895

**Published:** 2022-01-03

**Authors:** Mohammed Helboob, Haris Iftikhar, Mohammed Abdurabu, Shahzad Anjum, T. Suresh Kumar

**Affiliations:** 1 Emergency Medicine, Hamad Medical Corporation, Doha, QAT

**Keywords:** anticoagulation, bilateral, occlusion, popliteal artery, peripheral arterial disease

## Abstract

Peripheral arterial disease (PAD) is a common condition of the upper and lower extremities caused by atherosclerosis. It is often accompanied by symptomatic cardiovascular and cerebrovascular disease and is a major risk factor for amputation of an extremity. Timely diagnosis and intervention can prevent morbidity in these patients. We describe a case of a 48-year-old man with multiple co-morbidities who presented to our emergency department (ED) complaining of acute onset of bilateral leg pain. He was found to have bilateral acute popliteal artery occlusion confirmed by angiography of the lower limbs. Treatment was started early, right after reaching the provisional diagnosis.

Popliteal artery occlusion is quite common, but it becomes a rare diagnosis when it happens bilaterally. Detailed evaluation of the peripheral arterial circulation and an early diagnosis seem to be important in the ED management of these patients.

## Introduction

Peripheral arterial disease (PAD) is a common condition caused by atherosclerotic occlusive disease in the upper and lower extremities. Risk factors for atherosclerosis include smoking, diabetes, advancing age, hyperlipidemia, hypertension, male gender, and a family history of PAD [1]. These risk factors have been further characterized. Diabetes is more strongly associated with femoral, popliteal, and tibial PAD, whereas other risk factors (e.g. smoking and hypertension) are associated with proximal disease in the aorta and iliofemoral arteries. PAD can be accompanied by symptomatic cardiovascular and cerebrovascular diseases. It is one of the major risk factors for lower-extremity amputation [2].

Popliteal artery occlusion due to atherosclerosis is a common occurrence, but bilateral occlusion is extremely rare. We report a case of a 48-year-old man with multiple co-morbidities who presented to our ED and was diagnosed with bilateral popliteal artery occlusion.

## Case presentation

A 48-year-old Sudanese male, a smoker with a past medical history of obesity, type 2 diabetes mellitus, hypertension, and dyslipidemia, presented to the emergency department with sudden onset of bilateral lower leg and foot pain and left foot numbness. Symptoms started suddenly while he was climbing the stairs. The pain was severe initially, improved in the right leg after he took rest, but persisted in the left side with numbness of the left foot. The patient denied any history of intermittent claudication. His family history was not significant.

On examination, the patient appeared well but he had severe agonizing pain in both legs. His vital signs were stable. There was no hair loss, muscle or skin atrophy, and no evidence of chronic limb ischemia (CLI). The vascular examination showed intact and equal bilateral femoral and popliteal pulses, but impalpable dorsalis pedis artery bilaterally with bilateral cold feet. Although he had a subjective complaint of numbness, sensations were present in both feet. Each popliteal fossa exam was normal without any swelling, pulsatile mass, or deformity. The rest of his physical exam was unremarkable.

**Figure 1:**
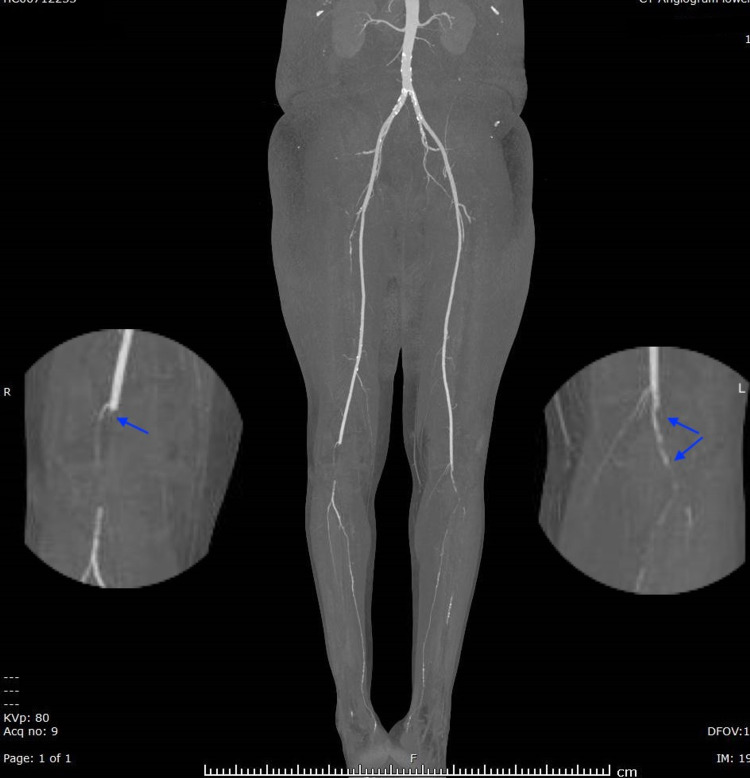
CT angiogram (anteroposterior view) showing right and left-sided popliteal artery occlusion (magnified image with blue arrows).

**Figure 2:**
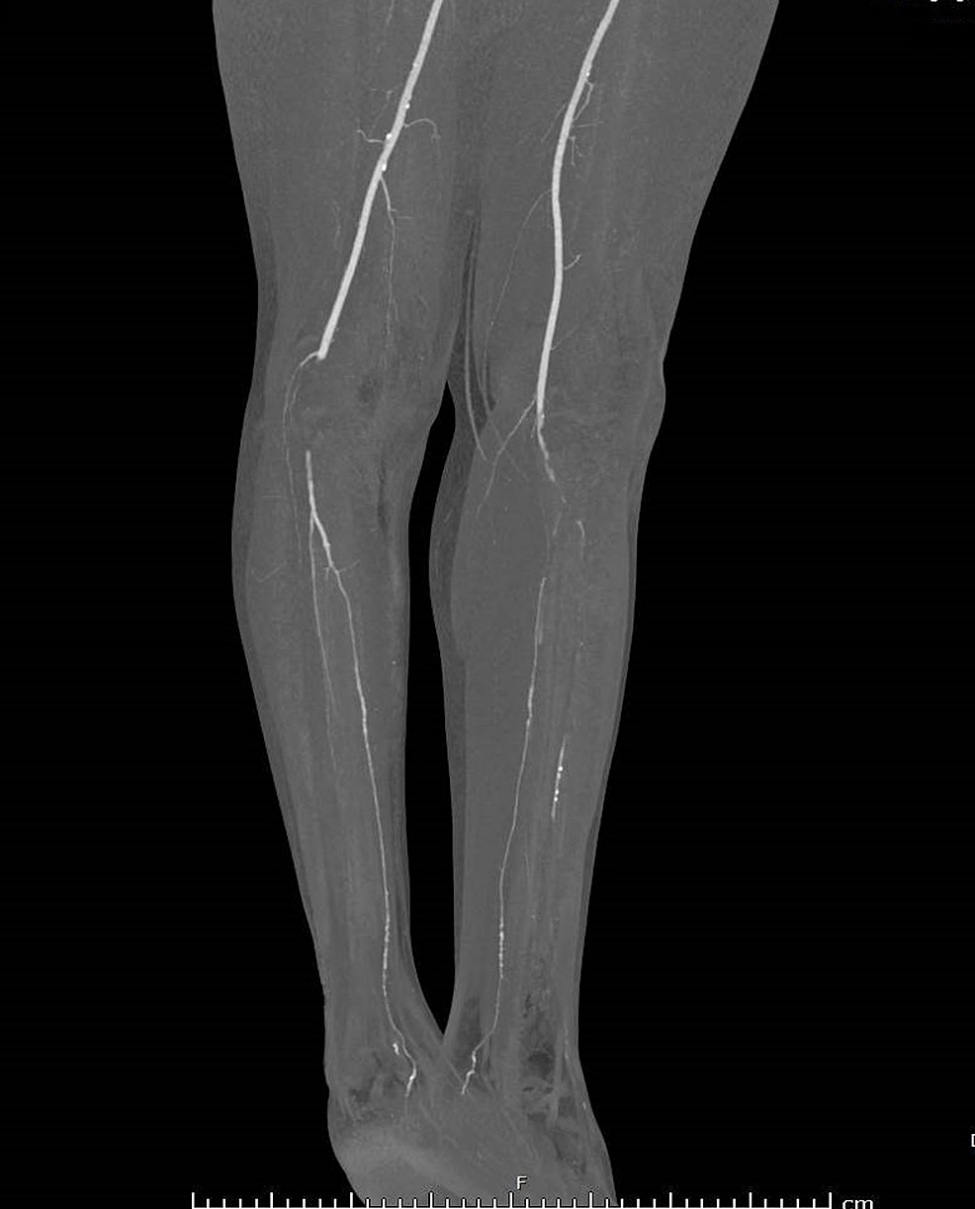
CT angiogram showing right and left-sided popliteal artery occlusion (lateral view).

Adequate analgesia was given to the patient. Bedside portable Doppler showed absent signal in the distal popliteal artery and below. Considering the diagnosis of bilateral acute limb ischemia (ALI) due to arterial occlusion, the vascular surgery team was consulted and the patient was immediately started on intravenous heparin in ED. Emergency CT angiogram of the abdomen, pelvis, and both lower limbs was performed. CT angiogram showed atherosclerotic changes noted in the visualized portion of the abdominal aorta and both common iliac arteries with no significant occlusion. The common femoral artery and superficial femoral arteries were patents on both sides. A 6 cm complete non-opacification in the right popliteal artery with distal reconstitution and distal occlusion of the anterior tibial artery was present. The rest of the branches were patent. However, on the left side, occlusion was starting at the distal part of the popliteal artery, extending to involve the tibioperoneal trunk and proximal part of the anterior tibial, peroneal, and posterior tibial arteries (Figures 1, 2), which confirmed the diagnosis of limb ischemia due to major artery occlusion. There was no evidence of popliteal aneurysm and popliteal band entrapment.

The patient had a normal complete blood count, but his liver and renal functions were deranged, showing creatinine of 141 umol/L (normal < 106), which were normal on previous occasions. The aspartate aminotransferase (AST) was 753 U/L (normal < 41) and alanine aminotransferase (ALT) was 127 U/L (normal < 40) with high prothrombin time (PT), partial thromboplastin time (PTT), and international normalized ratio (INR) (18, 32, and 1.7, respectively), indicating abnormal clotting. His thrombophilia screen, electrocardiogram, and chest X-ray were unremarkable.

The patient was admitted under vascular surgery care. He refused consent for urgent revascularization and was continued on heparin infusion and monitoring of PT, PTT, and INR values. The patient's symptoms improved with the treatment. The transthoracic echocardiography did not show cardiac thrombus as a source of emboli. The patient continued to receive three days of intravenous heparin infusion as a bridge overlapping with warfarin. The patient stayed in the hospital for a total of 13 days. His diabetes mellitus was optimized with medication adjustments. His renal and liver function tests became normal. He showed significant improvement in his symptoms. At discharge, his condition was stable with further follow-up in the vascular surgery clinic, warfarin clinic, and diabetes clinic.

## Discussion

The popliteal artery is one of the major arteries of the leg [3]. It starts as a continuation of the femoral artery and passes into the popliteal fossa superficial to the popliteus muscle. It terminates at the lower border of the popliteus by dividing into two terminal branches, which are the anterior and posterior tibial arteries. Through its course, the popliteal artery gives many branches. The popliteal artery gives articular branches to knee joints, muscular branches to hamstrings, adductor magnus, and triceps femoris, and the cutaneous branch to knee joint skin [4].

Atherosclerosis is the most common cause of popliteal artery occlusion or stenosis [3]. The pathogenesis of atherosclerosis usually starts with an endothelial injury, which then initiates a process of cellular mediators and overproduction, eventually producing fibrotic plaque. The plaque may calcify, fracture, or ulcerate, leading to hemorrhage and ultimately limiting blood flow or causing thrombosis of the vessel [1].

Lower limb pain is a common presentation to the ED. Acute limb ischemia (ALI) is not a common presentation. We need to be mindful and have a high degree of suspicion in any case of acute limb pain. This is often presented as unilateral. Causes of unilateral and bilateral leg pain include musculoskeletal (shin splints, stress fractures, myositis, and osteoarthritis), neurological (bilateral sciatica, cauda equina syndrome, and spinal canal stenosis), and vascular (atherosclerosis, vascular claudication, vasculitis, and thromboembolism) [5]. Other causes include cellulitis, psychogenic, and fibromyalgia. Atherosclerosis is a key pathology causing narrowing of vessels and distal ischemia. Increasing age, smoking, hypertension, and diabetes mellitus (DM) are risk factors for developing atherosclerosis. ALI affecting both limbs is infrequent. Acute ischemia of both limbs is possible if a proximal occlusion is present in the aorta or bilateral common iliac vessels [4]. Popliteal artery aneurysm is an important cause of acute unilateral or bilateral ischemia [6]. This case is unique as atherosclerotic changes with sudden occlusion below the knee at the popliteal fossa are present.

Clinical assessment of ALI should include duration of symptoms, pain intensity, and severity of motor and sensory deficit. The bedside exam should also include arterial and venous examination with a handheld Doppler. The absence of an arterial signal indicates that the limb is threatened. The loss of both arterial and venous signals means that the limb may be irreversibly damaged. The American Heart Association (AHA)/American College of Cardiology (ACC) guidelines for ALI recommend emergent evaluation by a vascular specialist if available. ED evaluation by a specialist should include limb viability and the possibility of salvage and does not need imaging. Definitive therapy should not be delayed. Even with rapid and effective revascularization, morbidity and mortality are high. Intravenous heparin is given to all patients acutely unless contraindicated [7].

The AHA 2005 classification scheme for femoropopliteal lesions recommends surgery for complete occlusion of popliteal artery extending to trifurcation (Table 1) [4].

**Table 1:** The AHA 2005 summary for preferred therapy for the femoropopliteal lesions. PTA = percutaneous transluminal angioplasty; AHA = American Heart Association.

Category	Description	Preferred therapy
1	Single stenosis < 5 cm, single occlusion < 3 cm, does not involve the superficial femoral artery origin or trifurcation	PTA
2	Single stenosis = 5-10 cm, single occlusion = 3-10 cm but not extending to trifurcation, heavily calcified stenosis < 5 cm, multiple lesions < 3 cm without continuous runoff	PTA
3	Occlusion = 3-10 cm extending to trifurcation, multiple lesions = 3-5 cm, single lesion > 10 cm	Surgery
4	Complete occlusion of the superficial femoral artery, popliteal artery, and trifurcation; severe diffuse disease with no segments	Surgery

AHA/ACC 2016 guidelines classify ALI into three categories. Category I is a viable limb that is not immediately threatened. Category IIa is marginally threatened but salvageable limb, if promptly treated. Category IIb is an immediately threatened limb that requires immediate intervention. Category III is an irreversibly damaged limb. For category I, revascularization should be performed on an urgent basis (within 6-24 hours). For category II, revascularization should be performed emergently (within six hours). The revascularization strategy can range from catheter-directed thrombolysis to surgical thrombectomy. However, our patient was only managed conservatively and has improved, and his symptoms resolved at the time of discharge [7].

ALI can be due to PAD or other conditions through either thrombotic or embolic mechanisms. Evaluation for embolic cause includes electrocardiogram to detect atrial fibrillation and evidence of myocardial infarction (MI). Echocardiography is useful to further determine whether there is a cardiac etiology for thromboembolism. Aziz et al. reported a case of bilateral popliteal artery occlusion due to thromboembolism from the left ventricle [8]. AHA/ACC recommends treatment of ALI should not be delayed for testing for the underlying cause like echocardiography because delay to revascularization is a major determinant of outcome [7].

Our PubMed and Google search did not reveal any other previous case report of bilateral popliteal artery occlusion due to thrombosis in any age group. Kapur et al. reported a case of bilateral acute lower limb ischemia secondary to aortic occlusion who underwent emergency bilateral ileo-femoral and aortic embolectomy [9]. Siau et al. reported a case with bilateral calf chronic compartment syndrome in an elderly male who presented with bilateral lower leg pain [10]. Abnormal soft tissues structures around the popliteal artery can cause unilateral or bilateral compression of the popliteal arteries. Popliteal artery entrapment syndrome (PAES) was first described in 1879 by Stuart due to abnormal embryologic development. PAES usually affects young athletes with well-developed calf muscles, without risk factors for atherosclerosis [11-14]. We have reported this rare case of bilateral popliteal occlusion due to atherosclerosis. The history was very atypical considering the absence of any previous claudication history or chronic features of limb ischemia. He was managed conservatively with anticoagulation and improved without any complications.

## Conclusions

Bilateral popliteal artery occlusion in a young patient is a unique diagnosis and needs reporting as we did not find any such case in the literature. The key learning is a thorough examination, having an open mind for differential diagnosis, and making a timely diagnosis. Otherwise, there is a risk of significant morbidity and impact on patient's safety.

## References

[1] Tendera M, Aboyans V, Bartelink ML (2011). ESC guidelines on the diagnosis and treatment of peripheral artery diseases: document covering atherosclerotic disease of extracranial carotid and vertebral, mesenteric, renal, upper and lower extremity arteries: the Task Force on the Diagnosis and Treatment of Peripheral Artery Diseases of the European Society of Cardiology (ESC). Eur Heart J.

[2] American Diabetes Association (2003). Peripheral arterial disease in people with diabetes. Diabetes Care.

[3] Shu J, Santulli G (2018). Update on peripheral artery disease: epidemiology and evidence-based facts. Atherosclerosis.

[4] Wright LB, Matchett WJ, Cruz CP, James CA, Culp WC, Eidt JF, McCowan TC (2004). Popliteal artery disease: diagnosis and treatment. Radiographics.

[5] Whitehouse WM, Wakefield TW, Graham LM (1983). Limb-threatening potential of arteriosclerotic popliteal artery aneurysms. Surgery.

[6] Shortell CK, DeWeese JA, Ouriel K, Green RM (1991). Popliteal artery aneurysms: a 25-year surgical experience. J Vasc Surg.

[7] Gerhard-Herman MD, Gornik HL, Barrett C (2017). 2016 AHA/ACC guideline on the management of patients with lower extremity peripheral artery disease: executive summary: a report of the American College of Cardiology/American Heart Association Task Force on Clinical Practice Guidelines. Circulation.

[8] Aziz F, Doddi S, Kallu S, Penupolu S, Alok A (2010). Myocardial infarction (MI) presenting as acute limb: an extremely rare presentation of MI. J Thorac Dis.

[9] Kapur P, Rakheja D, Amirkhan RH (2006). Acute-onset, bilateral lower extremity pain in a 30-year-old man. Lab Med.

[10] Siau K, O'Rourke KP, Khanna A, Laversuch CJ (2009). Bilateral calf chronic compartment syndrome in an elderly male: a case report. Cases J.

[11] Levien LJ (2003). Popliteal artery entrapment syndrome. Semin Vasc Surg.

[12] Stuart TP (1879). Note on a variation in the course of the popliteal artery. J Anat Physiol.

[13] Kamphuis D, Raber M, Meerwaldt R (2011). Case report: bilateral PAES as cause of lower leg pain in young athlete. Am Fam Physician.

[14] McAree BJ, O'Donnell ME, Davison GW, Boyd C, Lee B, Soong CV (2008). Bilateral popliteal artery occlusion in a competitive bike rider: case report and clinical review. Vasc Endovascular Surg.

